# Hepatocyte Endoplasmic Reticulum Stress Inhibits Hepatitis B Virus Secretion and Delays Intracellular Hepatitis B Virus Clearance After Entecavir Treatment

**DOI:** 10.3389/fmed.2020.589040

**Published:** 2021-02-04

**Authors:** Huan Chen, Maoyuan Mu, Qichuan Liu, Han Hu, Caiyun Tian, Guoyuan Zhang, Ying Li, Fangwan Yang, Shide Lin

**Affiliations:** Department of Infectious Diseases, Affiliated Hospital of Zunyi Medical University, Zunyi, China

**Keywords:** endoplasmic reticulum stress, HepG2215, thapsigargin, stearic acid, HBsAg, HBV DNA

## Abstract

**Background:** The aim of this study was to explore the effects of endoplasmic reticulum (ER) stress on hepatitis B virus (HBV) replication and the antiviral effect of entecavir (ETV).

**Methods:** Thapsigargin (TG) and stearic acid (SA) were used to induce ER stress in HepG2.2.15 cells and HepAD38 cells that contained an integrated HBV genome, while ETV was used to inhibit HBV replication. The expression levels of glucose-regulated protein 78 (GRP78) and phosphorylated eukaryotic translation initiation factor 2 subunit alpha (p-eIF2α) were measured by western blotting. Intracellular HBV DNA was determined by qPCR; HBsAg by western blotting; HBV RNA by real-time RT-qPCR; HBsAg and HBeAg in supernatants by enzyme-linked immunosorbent assay (ELISA); and HBV DNA in supernatants by qPCR.

**Results:** TG and SA induced ER stress in HepG2.2.15 cells and HepAD38 cells from 12 to 48 h post treatment. However, 4-phenylbutyric acid (PBA) partly alleviated the TG-induced ER stress. Moreover, TG inhibited HBsAg, HBeAg, and HBV DNA secretion from 12 to 48 h, while different concentrations of SA inhibited HBsAg and HBV DNA secretion at 48 h. TG promoted intracellular HBV DNA and HBsAg accumulation and the transcription of the HBV 3.5-kb mRNA and S mRNA. PBA treatment restored the secretion of HBsAg and HBV DNA. Finally, ER stress accelerated extracellular HBV DNA clearance but delayed intracellular HBV DNA clearance after ETV treatment.

**Conclusions:** Hepatocyte ER stress promoted intracellular HBV DNA and HBsAg accumulation by inhibiting their secretion. Our study also suggested that hepatocyte ER stress delayed intracellular HBV DNA clearance after ETV treatment.

## Introduction

Chronic hepatitis B virus (HBV) infection constitutes a global health concern. More than 240 million people are chronically infected with HBV, which puts them at great risk of developing end-stage liver diseases such as liver failure, cirrhosis, and liver cancer (1). The progression of patients with chronic HBV infection depends on a complex interaction between the host and the virus (2).

The HBV genome contains four partially overlapping open reading frames, named C, S, P, and X, from which 3.5-, 2.4-, 2.1-, and 0.7 kb-long RNAs, respectively, are transcribed. Translation of the 2.4- and 0.7-kb fragments produces the large envelope protein and the HBx protein, respectively. The 2.1-kb mRNA yields both the middle and small envelope proteins. The 3.5-kb long RNA, also called pregenomic RNA, is the only HBV transcript required for genome replication (1, 3).

The endoplasmic reticulum (ER) is an important cellular organelle and plays a major role in protein synthesis, folding, modification, and transport, as well as in lipid synthesis and the maintenance of calcium homeostasis. When ER homeostasis is disturbed under physical or pathological stimuli, such as the disruption of calcium homeostasis, the accumulation of unfolded or misfolded proteins, glucose starvation, and hypoxia, the unfolded protein response (UPR) is activated (4). ER stress can initiate the UPR *via* the binding of glucose-regulated protein 78 (GRP78) to unfolded or misfolded proteins and by activating three different sensors: protein kinase R-like endoplasmic reticulum kinase (PERK), inositol-requiring enzyme 1 (IRE1), and activating transcription factor 6 (ATF6).

ER stress is a common pathological phenomenon in patients with liver disease, including viral hepatitis, fatty liver disease, alcoholic liver disease, drug-induced hepatitis, and ischemic liver damage, among others (5, 6). Viruses, fatty acids, alcohol, and drugs can all induce ER stress in hepatocytes, which can damage these cells and lead to metabolic disorders.

HBV and hepatocytes have a complex and close interaction. The activation of HBV enhancers largely depends on cellular factors expressed in hepatocytes (7, 8). Transcription factors in hepatocytes such as CCAAT/enhancer-binding protein (C/EBP), cyclic-adenosine monophosphate-responsive element-binding protein (CREB), and several other nuclear factors have been found to play an important role in HBV enhancer activation (8, 9). In the HBV life cycle, secretory proteins, such as HBsAg and HBeAg, are folded and assembled in the ER of hepatocytes (10). However, how hepatocyte ER stress affects HBV replication, and the antiviral effect of nucleos(t)ide analogs, remains unknown. In this study, we employed thapsigargin (TG) and stearic acid (SA) as ER stress inducers in HBV-transfected HepG2.2.15 cells and HepAD38 cells to investigate the impact of ER stress on HBV replication and the antiviral efficacy of entecavir (ETV).

## Materials and Methods

### Reagents

RPMI 1640 was obtained from Thermo-Fisher Biochemical Products Co., Ltd (Beijing, China). TG, SA, and 4-phenylbutyric acid (PBA) were purchased from Sigma (St. Louis, MO, USA). Antibodies against GRP78, HBsAg, and beta-actin were purchased from Santa Cruz Biotechnology (Santa Cruz, CA, USA). The anti-phosphorylated eukaryotic translation initiation factor 2 alpha (p-eIF2α) antibody was purchased from Cell Signaling Technology (BioConcept, Allschwil, Switzerland). ETV was purchased from Solarbio Science & Technology Co., Ltd (Beijing, China). All other chemicals and reagents were obtained from Sigma (St. Louis, MO, USA).

### Cell Culture

HepG2.2.15 cells and HepAD38 cells with a stably integrated HBV genome were obtained from the cell bank of the Type Culture Collection of the Chinese Academy of Sciences (Shanghai, China). HepG2.2.15 cells and HepAD38 cells were cultured to 80–100% confluence. To investigate the effects of TG and SA on ER stress, HepG2.2.15 and HepAD38 cells were treated with TG (1 μM) for 12, 24, 36, and 48 h or SA (50, 100, or 200 μM) for 48 h. Similarly, PBA was used to alleviate ER stress and ETV to inhibit HBV replication. Briefly, HepG2.2.15 cells were pretreated with PBA (1 mM) for 2 h and then incubated with TG for 96 h, whereas ETV (10 μM) and TG (1 μM) were simultaneously administered for the same length of time. SA (0.017 g) was dissolved in 3 ml of 0.1 mM NaOH in a water bath at 100°C. Stock solutions were prepared by adding 3 ml of 40% fatty acid-free bovine serum albumin to each tube and incubating for 30 min at 55°C. The final SA concentration was 10 mM. All control conditions included the corresponding vehicles at the appropriate concentrations.

### Flow Cytometry

Apoptosis was determined using an Annexin V–fluorescein isothiocyanate (FITC)/propidium iodide (PI) apoptosis detection kit following the manufacturer's instructions. Briefly, 2 × 10^6^ cells were harvested and washed twice with precooled PBS and resuspended in 500 μl of binding buffer. Then, 5 μl of Annexin V–FITC and 5 μl of PI was added to each sample followed by incubation at room temperature in the dark for 10 min. Analysis was performed by flow cytometry (Beckman Coulter Gallios, USA) according to the manufacturer's specifications.

### Western Blotting

Cell lysates containing 40 μg of protein were resolved by sodium dodecyl sulfate–polyacrylamide gel electrophoresis (SDS–PAGE) using a 7–12.5% polyacrylamide gradient gel, and the fractioned proteins were subsequently transferred to polyvinylidene fluoride membranes (Millipore, Billerica, MA, USA). After being blocked with Tris-buffered saline containing 5% dry milk and 0.1% Tween 20 for 1 h, the membranes were blotted with the corresponding antibodies. The following primary antibodies were used: rabbit anti-human GRP78 (sc-376768, 1:10,000), anti-p-eIF-2α (3398, 1:10,000), mouse anti-human β-actin (sc-58673, 1:10,000), and anti-HBsAg (sc-53300, 1:1,000). The secondary antibodies were horseradish peroxidase-conjugated goat anti-rabbit IgG and horseradish peroxidase-conjugated goat anti-mouse IgG. The membranes were developed using a chemiluminescence detection system and then exposed to Kodak BioMax Light Film (Rochester, NY, USA). The band intensity for each protein was measured densitometrically and normalized to the level of β-actin.

### Quantification of HBsAg and HBeAg

HepG2.2.15 and HepAD38 cells were cultured at a density of 1 × 10^4^ cells per well in RPMI 1640 medium. After drug treatment, the levels of HBsAg and HBeAg in the supernatants were measured using an enzyme-linked immunosorbent assay (ELISA) (Kehua Bio-engineering Corp.) according to the manufacturer's recommendations.

### DNA and RNA Isolation, Reverse Transcription, and Real-Time Polymerase Chain Reaction

Total DNA was extracted using a QIAamp DNA Mini Kit (Qiagen). Total RNA was extracted from HepG2.2.15 cells using a TaKaRa MiniBEST Universal RNA Extraction Kit (9767, Takara). The HBV DNA content was quantified by real-time qPCR using SYBR Green I. The primers used to amplify the HBV DNA were as follows: 5′-GTTGCCCGTTTGTCCTCTAATTC-3′ and 5′-GGAGGGATACATAGAGGTTCCTT-3′. Quantitative PCR was performed at 95°C for 30 s, 40 cycles of 95°C for 5 s, and 60°C for 34 s, followed by melting curve analysis, according to the instrument documentation. The RNA samples were first reverse transcribed into cDNA using the PrimeScript RT Reagent Kit (DRR037A, Takara). The products were then subjected to qPCR using the following primers: HBV-S mRNA, 5′-CTAGGACCCCTGCTCGTG-3′ and 5′-GATGAGGCATAGCAGCAG-3′; HBV 3.5-kb mRNA, 5′-CTCAATCTCGGGAATCTCAATGT-3′ and 5′-TGGATAAAACCTAGCAGGCATAAT-3′; and GADPH mRNA, 5′-CGACCACTTTGTCAAGCTCA-3′ and 5′-ACAGCCTGGATAGCAACG-3′. The relative HBV DNA and mRNA levels were determined using the comparative (2^−ΔΔCT^) method, as previously described (11).

### Statistical Analysis

The results were expressed as means ± standard deviation for “*n*” independent observations. One-way ANOVA was used to determine the statistical differences between the mean values for the groups. The level of significance was set at *P* < 0.05.

## Results

### Thapsigargin and Stearic Acid Treatment Induced Endoplasmic Reticulum Stress in HepG2.2.15 and HepAD38 Cells, Which Was Alleviated by Exposure to 4-Phenylbutyric Acid

**Figure 1:**
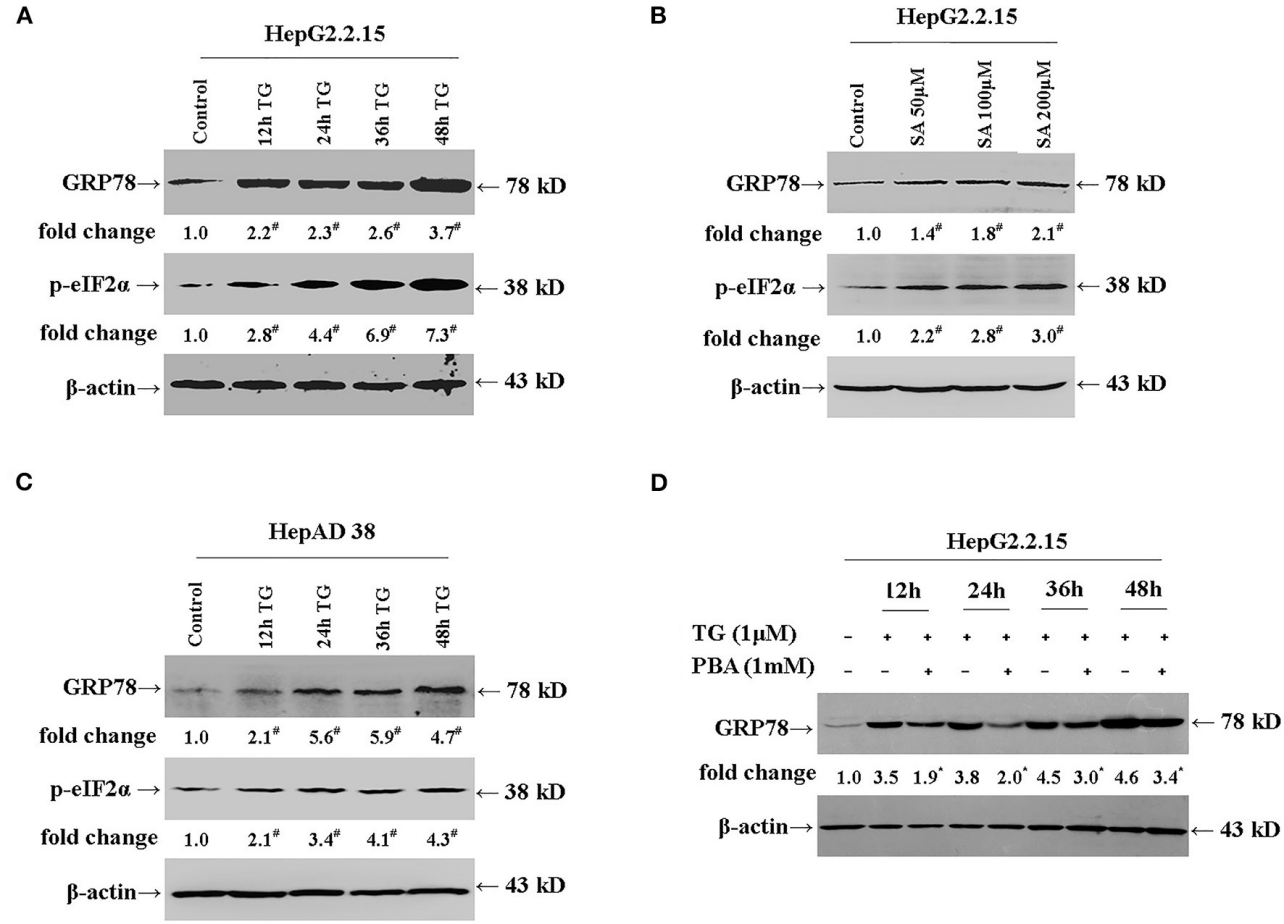
TG and SA induced ER stress in HepG2.2.15 and HepAD38 cells, which was alleviated by PBA treatment. HepG2.2.15 and HepAD38 cells were treated with 1 μM of TG for 12, 24, 36, and 48 h or 50, 100, and 200 μM of SA for 48 h. The expression of GRP78 and p-eIF2α was assessed by western blotting. Representative blots from three independent experiments are shown. The results of the densitometric analysis are presented as fold-changes compared with control or TG. **(A)** GRP78 and p-eIF2α expression in HepG2.2.15 cells after TG treatment. ^#^*P* < 0.05 vs. control. **(B)** GRP78 and p-eIF2α expression in HepG2.2.15 cells following SA treatment. ^#^*P* < 0.05 vs. control. **(C)** GRP78 and p-eIF2α expression in HepAD38 cells after TG treatment. ^#^*P* < 0.05 vs. control. **(D)** GRP78 expression in HepG2.2.15 cells after TG or PBA (1 mM) + TG treatment. *P < 0.05 vs. the TG group. ER, endoplasmic reticulum; GRP78, glucose-regulated protein 78; PBA, 4-phenylbutyric acid; p-eIF2α, phospho-eukaryotic translation initiation factor 2 alpha; SA, stearic acid; TG, thapsigargin.

We first investigated whether TG and SA induced ER stress in HepG2.2.15 and HepAD38 cells. As shown in Figures 1A,B, TG significantly induced GRP78 and p-eIF2α expression from 12 to 48 h post treatment at the 1-μM concentration in HepG2.2.15 cells. Similarly, SA markedly induced GRP78 and p-eIF2α expression at 48 h at the concentrations of 50, 100, and 200 μM in HepG2.2.15 cells. At 100 μM, SA significantly induced GRP78 expression from 24 to 72 h, as did TG at 24 h at the concentrations of 0.5, 1, and 2 μM in HepG2.2.15 cells (Supplementary Figures 1, 2). Furthermore, 1 μM of TG significantly induced GRP78 and p-eIF2α expression from 12 to 48 h post treatment in HepAD38 cells (Figure 1C). Our results demonstrated that both TG and SA could induce ER stress and activate the UPR in HepG2.2.15 cells and HepAD38 cells.

Next, we investigated whether PBA could alleviate the ER stress induced by TG (Figure 1D). We found that at the 1-mM concentration, PBA could significantly inhibit the TG-mediated induction of GRP78 expression, demonstrating that PBA could alleviate TG-induced ER stress.

We next evaluated the effect of TG and SA on the apoptosis and viability of HepG2.2.15 cells. At the concentration of 1 μM, TG treatment did not lead to a marked increase in the rate of apoptosis from 12 to 48 h post treatment, and a similar effect was observed for SA treatment (50 and 100 μM) at 48 h (Supplementary Figure 3).

### Thapsigargin and Stearic Acid Inhibited the Secretion of HBsAg, HBeAg, and Hepatitis B Virus DNA

**Figure 2:**
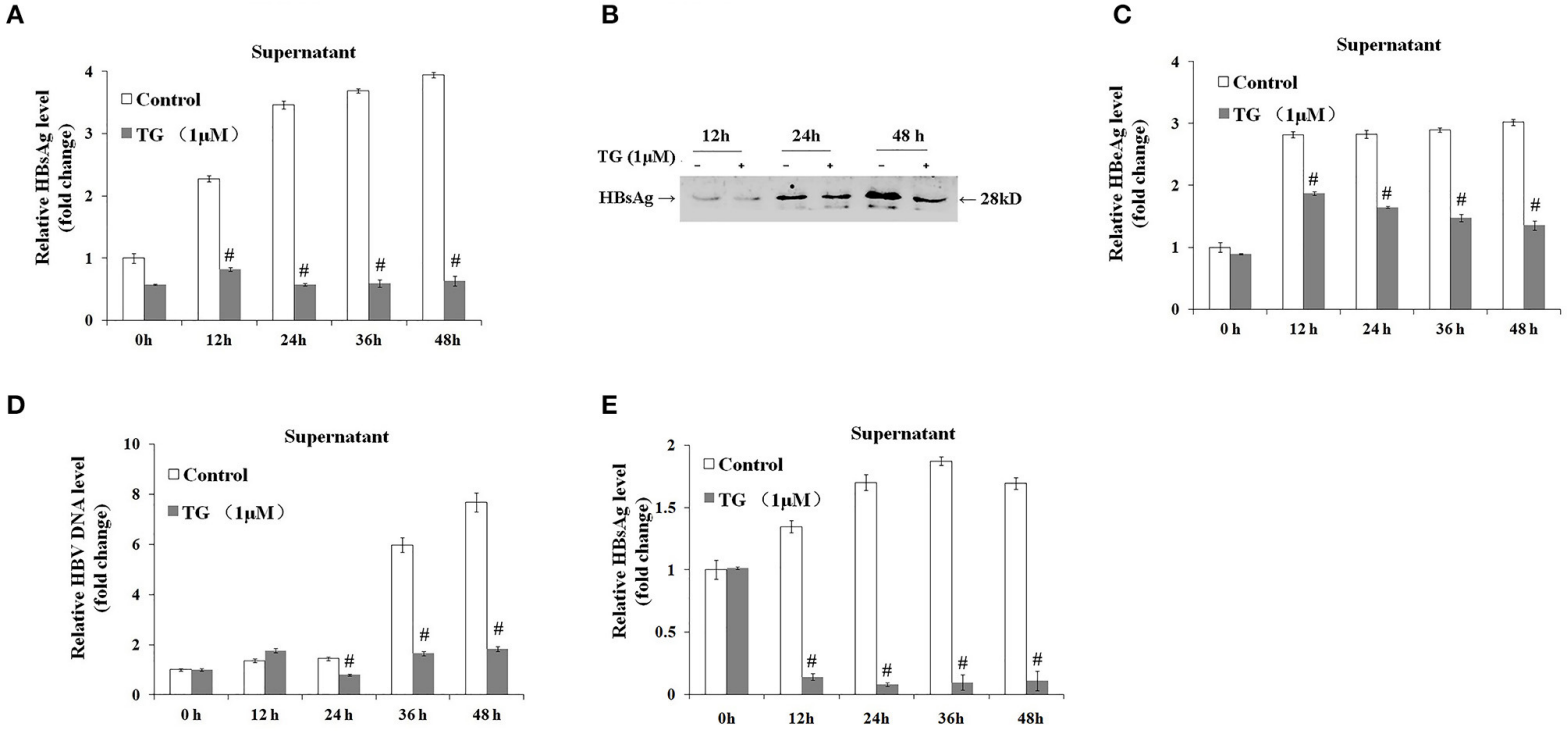
Thapsigargin (TG) inhibited HBsAg, HBeAg, and hepatitis B virus (HBV) DNA secretion in HepG2.2.15 cells and HepAD38 cells. HepG2.2.15 cells and HepAD38 cells were treated with 1 μM of TG for 12, 24, 36, and 48 h. The supernatant levels of HBsAg and HBeAg were determined by ELISA or western blotting, while those of HBV DNA were determined by qPCR. **(A)** HBsAg levels as determined by ELISA in HepG2.2.15 cells. **(B)** HBsAg levels as determined by western blotting in HepG2.2.15 cells. **(C)** HBeAg levels as determined by ELISA in HepG2.2.15 cells. **(D)** HBV DNA levels as determined by qPCR in HepG2.2.15 cells. **(E)** HBsAg levels as determined by western blotting in HepAD38 cells. The results are presented as fold-changes compared with the control. Histograms represent the means ±*SD* of three independent experiments. ^#^*P* < 0.05 vs. the control.

**Figure 3:**
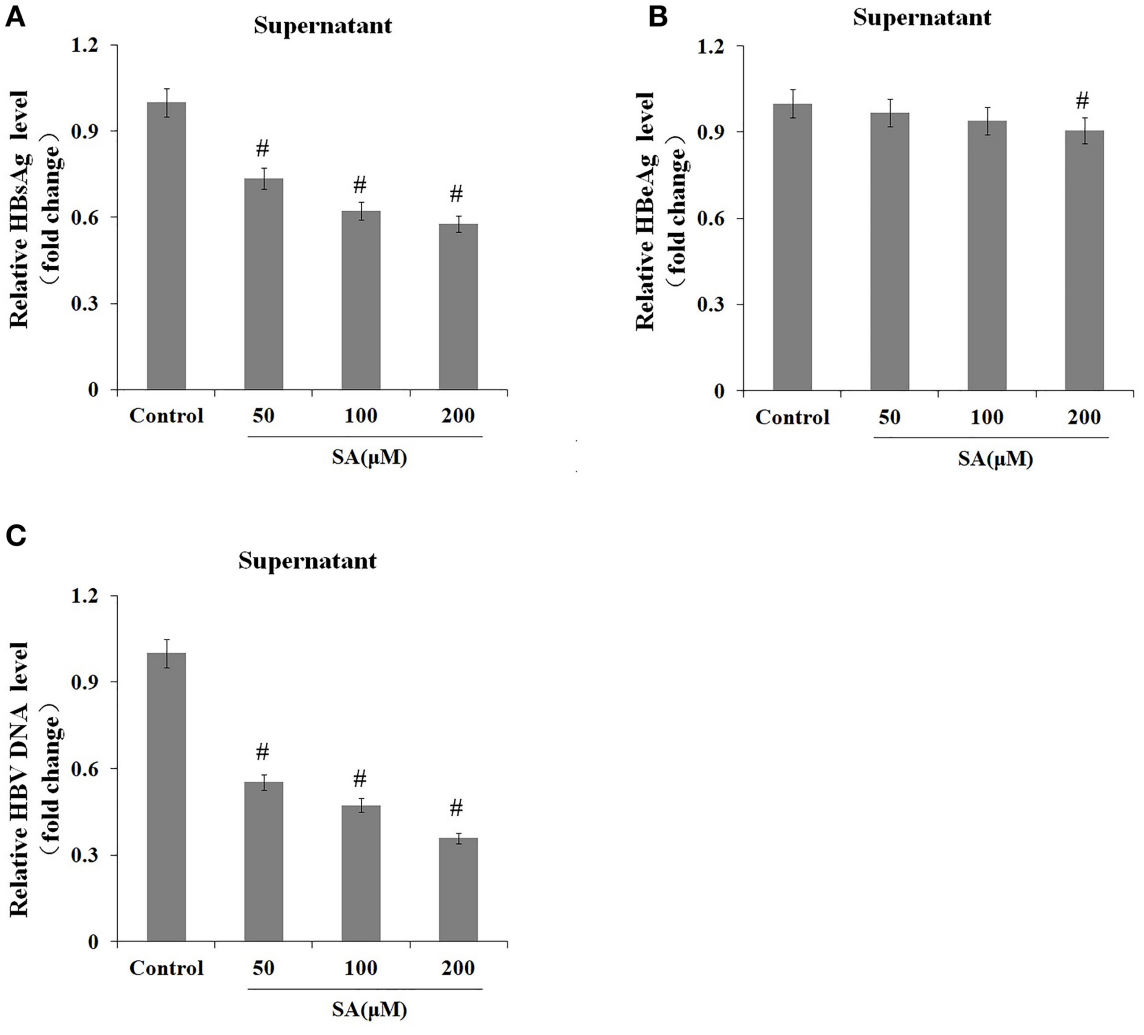
Stearic acid (SA) inhibited the secretion of HBsAg and hepatitis B virus (HBV) DNA in HepG2.2.15 cells. HepG2.2.15 cells were treated with 50, 100, and 200 μM of SA for 48 h. The supernatant levels of HBsAg and HBeAg were determined by ELISA, while those of HBV DNA were determined by qPCR. **(A)** HBsAg levels as determined by ELISA. **(B)** HBeAg levels as determined by ELISA. **(C)** HBV DNA levels as determined by qPCR. The results are presented as fold-changes compared with the control. Histograms represent the means ±*SD* of three independent experiments. ^#^*P* < 0.05 vs. the control.

We subsequently explored the effect of TG (1 μM) and SA (50, 100, and 200 μM) on the secretion of HBsAg, HBeAg, and HBV DNA in HepG2.2.15 supernatants. As shown in Figures 2, 3, exposure to TG significantly inhibited the secretion of HBsAg, HBeAg, and HBV DNA from 12 to 48 h post treatment. Similarly, treatment with 50, 100, and 200 μM of SA also greatly suppressed the secretion of HBsAg and HBV DNA at 48 h. We further found that TG significantly inhibited the secretion of HBsAg from 12 to 48 h post treatment in HepAD38 cells (Figure 2C). Western blotting results confirmed that TG significantly inhibited the secretion of HBsAg from 12 to 48 h. However, SA treatment did not significantly affect the secretion of HBeAg, at the concentration of either 50 or 100 μM (Figure 3). At 100 μM, SA also significantly inhibited the secretion of HBsAg and HBV DNA from 24 to 72 h, as did TG at 24 h at the concentrations of 0.5, 1, and 2 μM in HepG2.2.15 cells (Supplementary Figures 4–7).

### The Effects of Thapsigargin on the Transcription of Hepatitis B Virus DNA and Intracellular HBsAg and Hepatitis B Virus DNA Levels

**Figure 4:**
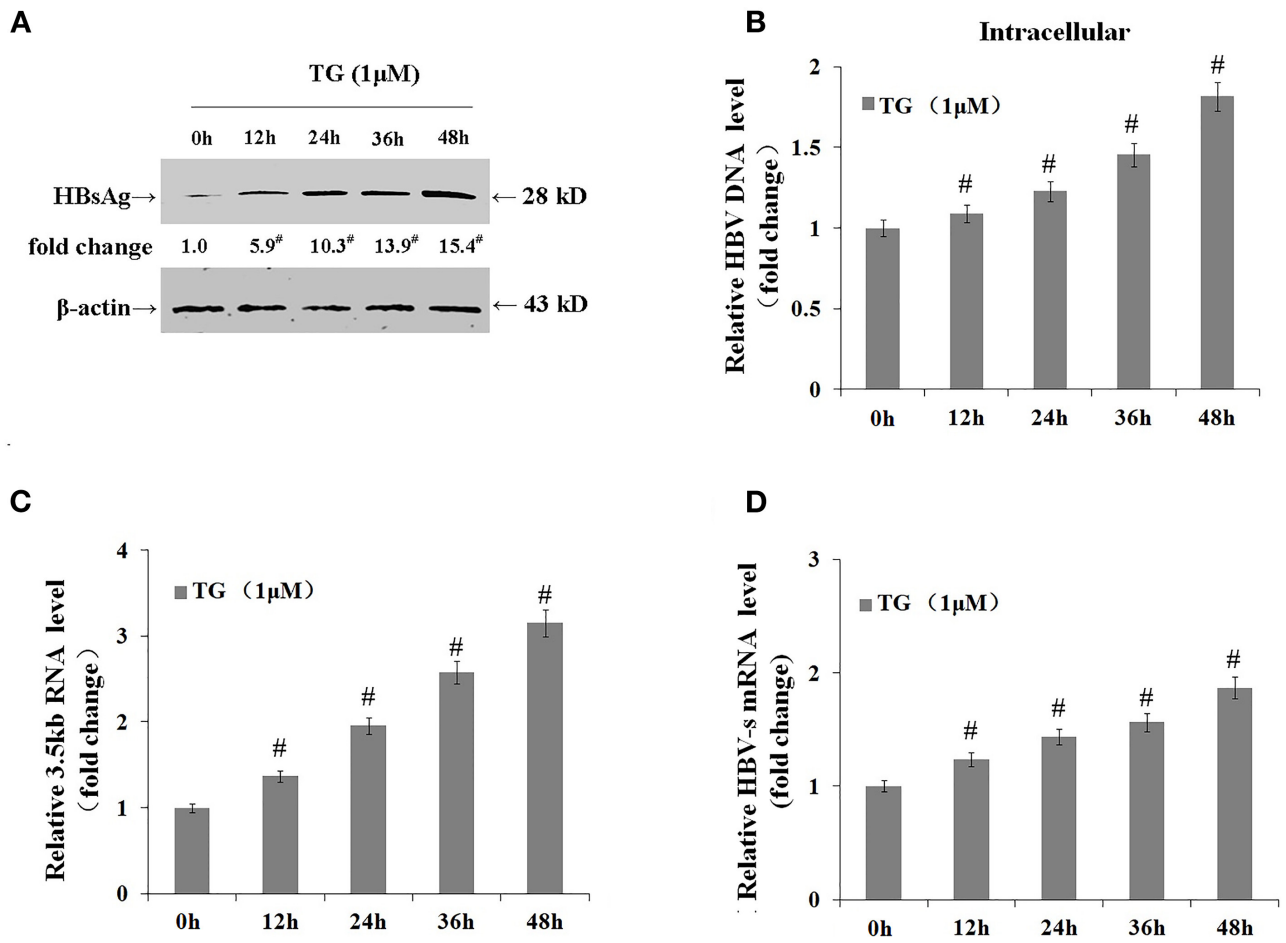
Thapsigargin (TG) promoted hepatitis B virus (HBV) DNA transcription and the accumulation of HBV DNA and HBsAg in HepG2.2.15 cells. HepG2.2.15 cells were treated with 1 μM of TG for 12, 24, 36, and 48 h. Intracellular HBsAg, HBV DNA, and HBV RNA levels were determined by western blotting, qPCR, and real-time RT-qPCR, respectively. The results are presented as fold-changes compared with the control. Histograms represent the means ±*SD* of three independent experiments. ^#^*P* < 0.05 vs. control. **(A)** HBsAg levels as determined by western blotting. Representative blots from three independent experiments are shown. **(B)** HBV DNA levels as determined by qPCR. **(C)** HBV 3.5-kb mRNA levels as determined by RT-qPCR. **(D)** HBV S mRNA levels as determined by RT-qPCR.

We further explored the effects of TG on the expression levels of the 3.5-kb mRNA and S mRNA as well as on the intracellular levels of HBsAg and HBV DNA. As shown in Figure 4, TG significantly upregulated the expression of the HBV 3.5-kb mRNA fragment and S mRNA and increased the intracellular levels of HBsAg and HBV DNA. Western blotting confirmed that the intracellular level of HBsAg was significantly increased by TG from 12 to 48 h post treatment.

### Thapsigargin Inhibited the Secretion of HBsAg and Hepatitis B Virus DNA *via* the Induction of Endoplasmic Reticulum Stress

**Figure 5:**
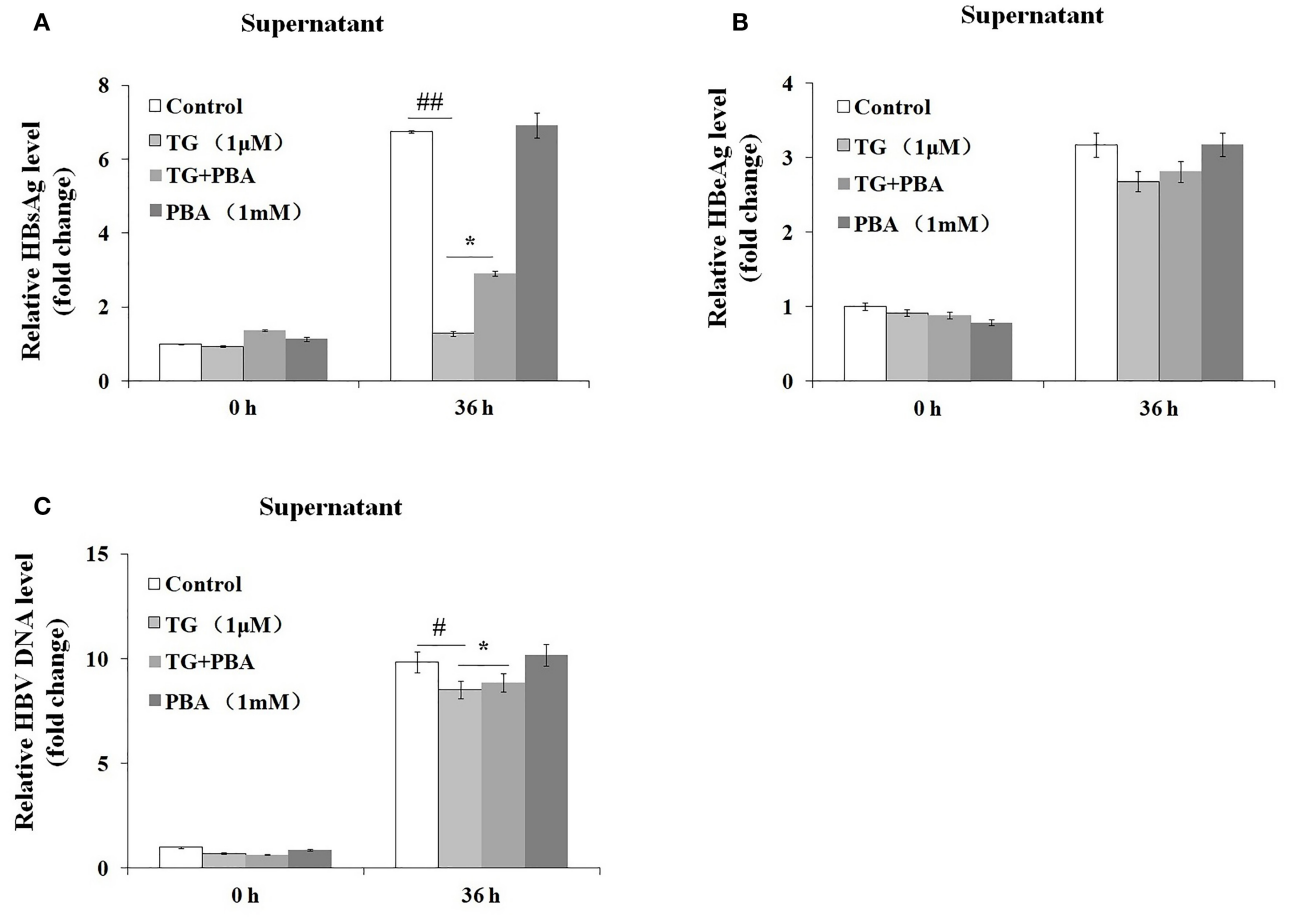
4-Phenylbutyric acid (PBA) pretreatment restored the secretion of hepatitis B virus (HBV) DNA, HBsAg, and HBeAg in HepG2.2.15 cells. HepG2.2.15 cells were treated with 1 μM of thapsigargin (TG) with or without 1 mM of PBA for 12, 24, 36, and 48 h. The supernatant levels of HBsAg and HBeAg were determined by ELISA, while those of HBV DNA were determined by qPCR. The results are presented as fold-changes compared with the control. Histograms represent the means ±*SD* of three independent experiments. ^#^*P* < 0.05 vs. the control ^*##*^*P* < 0.01, ^#^*P* < 0.05 vs. the control; ^*^P < 0.05. **(A)** HBsAg and **(B)** HBeAg levels as determined by ELISA. **(C)** HBV DNA levels as determined by qPCR.

We then investigated whether the inhibitory effects of TG on HBsAg and HBV DNA secretion were exerted through the induction of ER stress. As shown in Figure 5, the secretion of HBsAg and HBV DNA was significantly restored by PBA at 36 h post treatment. Similarly, HBeAg levels in the supernatant were also partially restored. These results strongly suggested that TG inhibited HBsAg, HBeAg, and HBV DNA secretion through the induction of ER stress.

### Thapsigargin Delayed Intracellular Hepatitis B Virus Clearance After Entecavir Treatment

**Figure 6:**
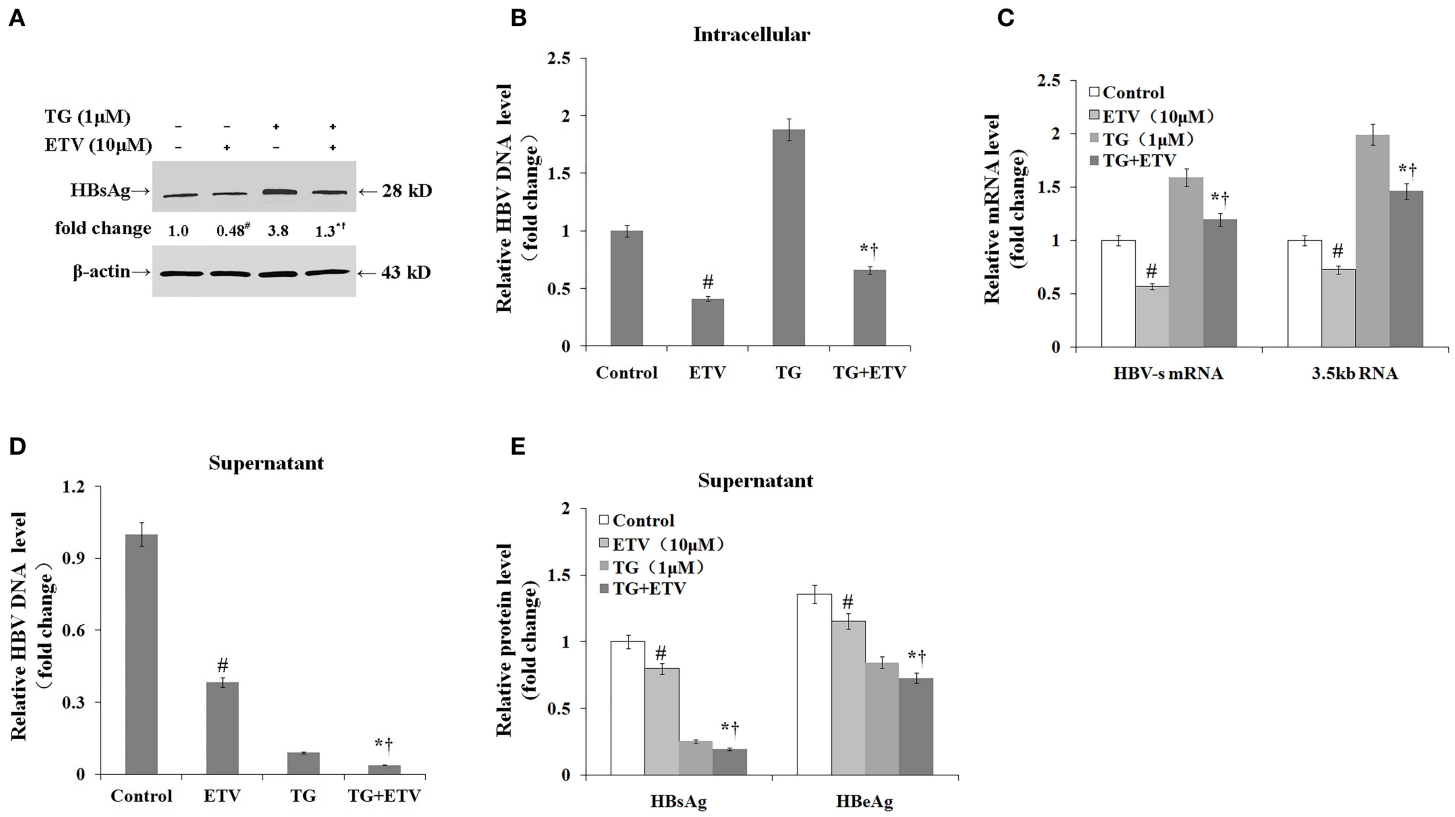
Thapsigargin (TG) delayed intracellular hepatitis B virus (HBV) clearance after entecavir (ETV) treatment. HepG2.2.15 cells were treated with 1 μM of TG with or without 10 μM of ETV for 96 h. The supernatant levels of HBsAg and HBeAg were determined by ELISA, while those of HBV DNA were determined by qPCR. Intracellular HBsAg, HBV DNA, and HBV RNA levels were determined by western blotting, qPCR, and RT-qPCR, respectively. The results are presented as fold-changes compared with the control or TG group. Histograms represent the means ±*SD* of three independent experiments. ^#^*P* < 0.05 vs. the control; *P < 0.05 vs. the TG group; ^†^*P* < 0.05 vs. the ETV group. **(A)** Intracellular HBsAg levels as determined by western blotting. Representative blots from three independent experiments are shown. **(B)** Intracellular HBV DNA levels as determined by qPCR. **(C)** Intracellular HBV RNA levels as determined by RT-qPCR. **(D)** HBV DNA levels in supernatants as determined by qPCR. **(E)** HBsAg and HBeAg levels in supernatants as determined by ELISA.

Finally, we assessed whether the TG-induced ER stress impaired the antiviral effect of ETV. After 96 h of ETV treatment, HBV replication was significantly inhibited (Figure 6). TG treatment significantly delayed the intracellular clearance of HBV DNA, HBsAg, and HBV RNA; in contrast, TG treatment significantly accelerated the extracellular clearance of HBV DNA, HBsAg, and HBeAg.

## Discussion

HBV assembly and HBsAg synthesis and secretion are highly dependent on ER function (10, 12). Each of the HBV envelope proteins is cotranslationally inserted into the ER membrane. The middle and small proteins have single transmembrane domains, whereas the large protein has a unique dual transmembrane conformation topology in the ER. The large protein is modified by myristoylation, but this is dispensable for virion formation (13–15). Host chaperones, such as heat shock protein 70 (HSP70) and GRP78/Bip, play an important role in the correct folding of envelope proteins and the maintenance of the dual topology of the large protein in the ER (16, 17). The synthesis of HBeAg is also closely associated with the ER (18, 19). As HBV replication and antigen secretion *in vivo* are both dependent on a complex interaction between the host and the virus, such as the activation of immune cells and the regulation of cytokine levels, it is difficult to identify the direct effect of ER stress on HBV replication *in vivo*. In this study, we used TG and SA to induce ER stress in HepG2.2.15 cells containing an integrated HBV genome and HepAD38 cells, and the results obtained mostly reflect the direct effect of ER stress on HBV replication.

In this study, we found for the first time that hepatocyte ER stress greatly suppressed HBsAg and HBV DNA secretion and increased the intracellular levels of HBsAg and HBV DNA. How ER stress affects HBV replication remains unknown. Several studies have reported that the accumulation of mutated HBsAg in hepatocytes results in ER stress and the activation of the UPR both *in vitro* and *in vivo* (20–22). However, the results regarding the effect of hepatocyte ER stress on HBV replication have been contradictory, and several different mechanisms have been suggested to be involved. Xu et al. reported that hepatocyte ER stress induced by the retention of the large protein of the HBV can activate the S promoter; increase the synthesis of the middle and small proteins to restore the proportions of the large, middle, and small proteins in cells; and maintain the assembly of HBV particles (23). Additionally, the authors also found that the transcriptional activation of the S promoter was cell type-restricted and was mediated through the IRE1α/X-box binding protein 1 (XBP1) pathway. A different study demonstrated that, in HBV-infected hepatoma cells, the ER stress-associated degradation pathway was activated, leading to the degradation of the HBV envelope protein and the consequent inhibition of HBV replication (24). Cisplatin-induced ER stress in hepatocytes has also been found to evoke HBV reactivation *via* the peroxisome proliferator-activated receptor gamma coactivator 1 alpha (PPARGC1A) signaling pathway (25). GRP78 is a master ER stress regulator, the expression of which is significantly upregulated during ER stress. Several studies have demonstrated that GRP78 expression can inhibit HBV DNA replication (26–28). We do not know the reasons for these differing results. In this study, we used TG and SA to induce ER stress, and we found that both inhibited HBV DNA and HBsAg secretion, while TG also promoted intracellular HBV DNA and HBsAg accumulation. PBA is a low-molecular-weight chemical chaperone that effectively prevents misfolded protein aggregation and alleviates ER stress. After PBA administration, HBV DNA and HBsAg secretion were partly restored. These results demonstrated that the inhibitory effects of TG and SA on HBV DNA and HBsAg secretion were at least partly dependent on the induction of ER stress.

The ER is the largest Ca^2+^ store in hepatocytes. Ca^2+^ release from the ER is a common mechanism underlying the occurrence and progression of ER stress following both physical and pathological cues. TG induces ER stress by irreversibly inhibiting sarcoplasmic/ER Ca^2+^-ATPase, which blocks the reabsorption of calcium from the cytosol to the ER and leads to an increase in the cytosolic Ca^2+^ concentration (29). Cytosolic Ca^2+^ can stimulate the Pyk2/Src kinase signal transduction pathway, thereby activating HBV reverse transcription and DNA replication (30). However, one study reported that HBV DNA replication was inhibited following the blocking of the ER Ca^2+^-ATPase (12). How SA, a saturated fatty acid, induces ER stress remains unclear. High saturated fatty acid concentrations have been found to impair ER structure and function (31). In our study, both TG and SA inhibited the secretion of HBV DNA and HBsAg. However, these effects were partially attenuated following PBA-mediated alleviation of ER stress. These results further supported that the inhibition of HBV DNA and HBsAg secretion by TG and SA was partly mediated *via* the induction of ER stress. However, whether TG promoted the transcription of both the HBV 3.5-kb mRNA fragment and S mRNA *via* an increase in the cytosolic Ca^2+^ concentration warrants further study.

Another major finding of this study was that, although ETV displayed high antiviral efficacy in the presence of TG, treatment with TG accelerated extracellular HBV DNA, HBsAg, and HBeAg clearance but delayed the clearance of intracellular HBV DNA, HBsAg, and HBV mRNA. These results suggested that hepatocyte ER stress delayed intracellular HBV clearance. The intracellular HBV DNA and HBsAg accumulation induced by TG exposure may partly explain this result. However, the mechanism underlying the impaired HBsAg and HBV DNA secretion and the enhanced transcription of the HBV 3.5-kb mRNA and S mRNA under ER stress needs further clarification. Additionally, given that ER stress is a common pathological phenomenon in patients with liver disease, the clinical significance of the delayed intracellular HBV clearance under ER stress after ETV treatment should also be explored.

In conclusion, this study is the first to report that hepatocyte ER stress promotes intracellular HBV DNA and HBsAg accumulation by inhibiting HBV DNA and HBsAg secretion. Our study also suggested that hepatocyte ER stress delays intracellular HBV DNA clearance after ETV treatment.

## Data Availability Statement

The original contributions presented in the study are included in the article/Supplementary Materials, further inquiries can be directed to the corresponding author/s.

## Author Contributions

SL designed the experiments and wrote the manuscript. HC, MM, QL, HH, and GZ performed the experiments. YL and FY revised the manuscript and analyzed the data. All authors read and approved the final manuscript.

## Conflict of Interest

The authors declare that the research was conducted in the absence of any commercial or financial relationships that could be construed as a potential conflict of interest.
