# Supplementary material for: Hepatocyte Endoplasmic Reticulum Stress Inhibits Hepatitis B Virus Secretion and Delays Intracellular Hepatitis B Virus Clearance After Entecavir Treatment
Source: Front Med (Lausanne). 2021 Feb 4;7:589040. doi: 10.3389/fmed.2020.589040 (PMC7890007; doi:10.3389/fmed.2020.589040)
Supplement: Supplementary file 1 [file Data_Sheet_1.PDF]

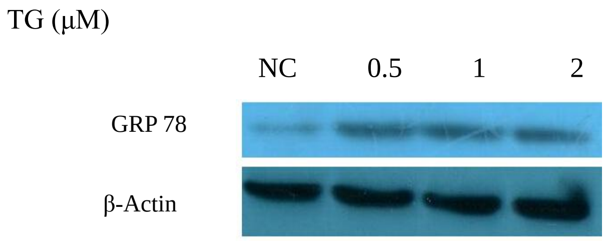

Supplement figure 1 Different concentration of TG induced GRP 78 expression at 24 hours inHepG2.2.15 cells. The expressions of GRP78 was assessed by western blotting. GRP78: glucose-regulated protein 78; TG: thapsigargin.

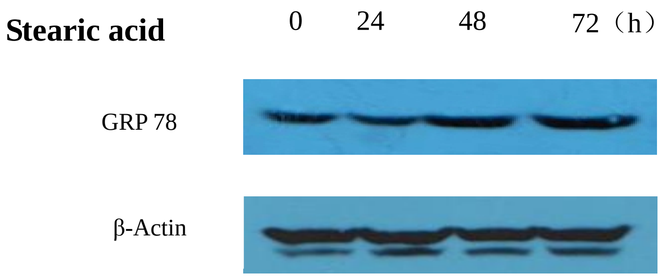

Supplement figure 2 Stearic acid induced GRP 78 expression from 24 to 72 hours in HepG2.2.15 cells. The expressions of GRP78 was assessed by western blotting.GRP78: glucose-regulated protein 78.

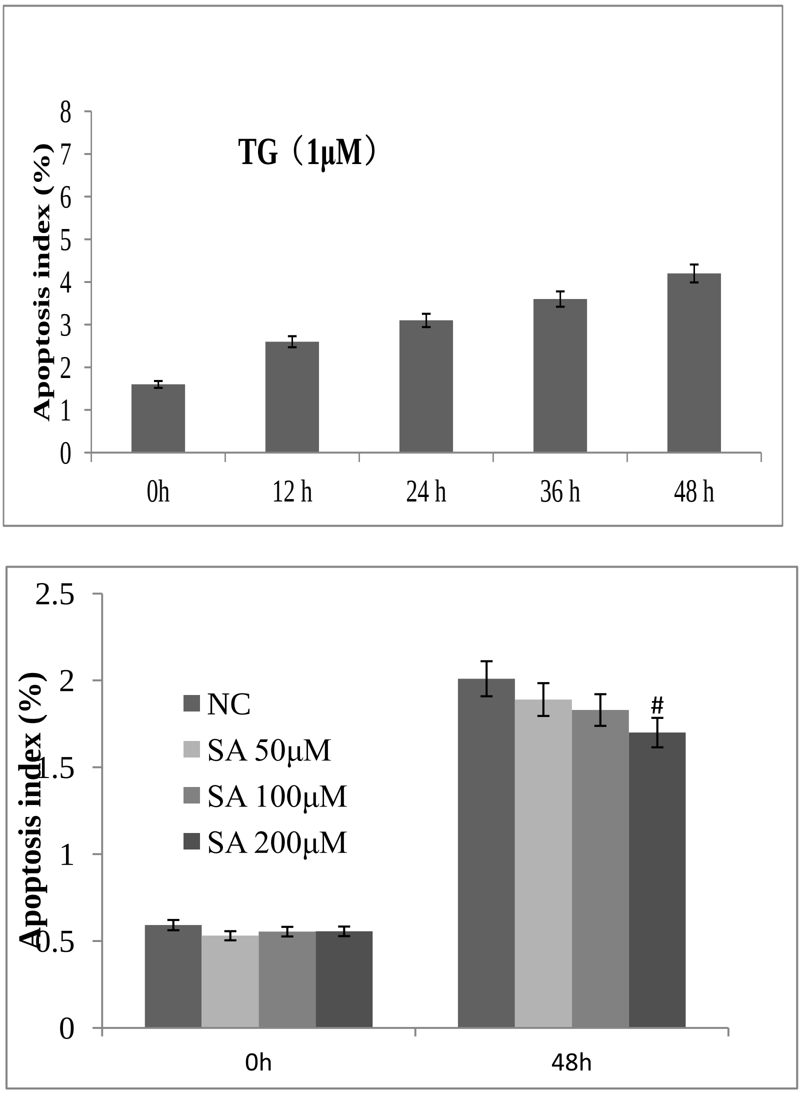

Supplement figure 3 The L02 cells were stimulated with 1μM TG or SA for different durations as indicated. Cell viability was determined by the method of Flow Cytometry. Histograms represent the means ± SD of three independent experiments. #P < 0.05 versus the control. NC, normal control; SA, Stearic acid; TG, thapsigargin.

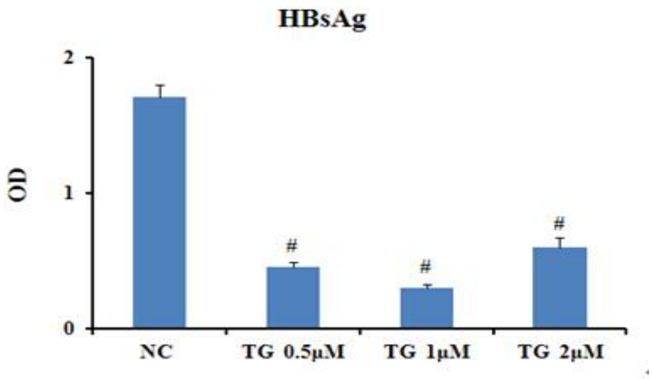

Supplement figure 4 Different concentration of thapsigargin inhibited HBsAg secretion at 24 hours in HepG2.2.15 cells. HBsAg in supernatants was determined by the methods of enzyme-linked immunosorbent assay. NC, normal control. # $P < 0.05$ versus NC.

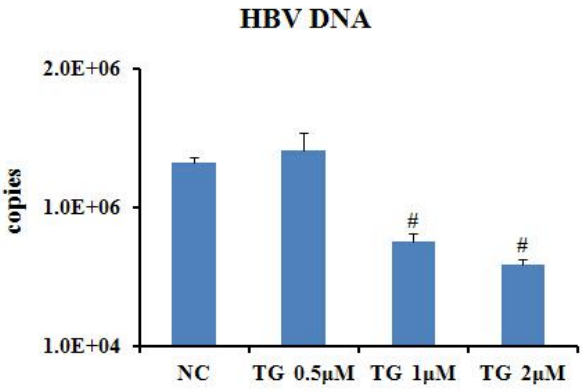

Supplement figure 5 Different concentration of thapsigargin inhibited HBVDNA secretion at 24 hours in HepG2.2.15 cells. HBV DNA in supernatants was determined by real-time polymerase chain reaction assay. NC, normal control. # $P < 0.05$ versus NC.

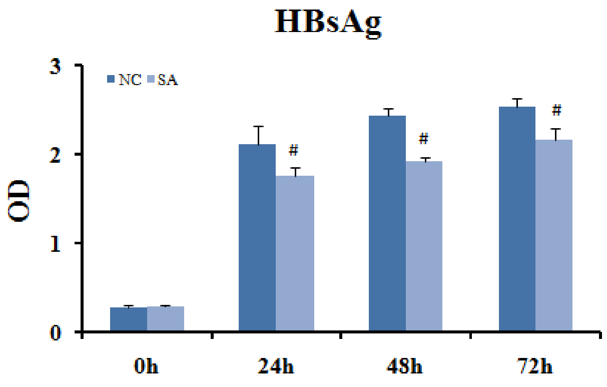

Supplement figure 6 Stearic acid inhibited HBsAg secretion from 24 to 72 hours in HepG2.2.15 cells. HBsAg in supernatants was determined by the methods of enzyme-linked immunosorbent assay. NC, normal control. # $P < 0.05$ versus NC.

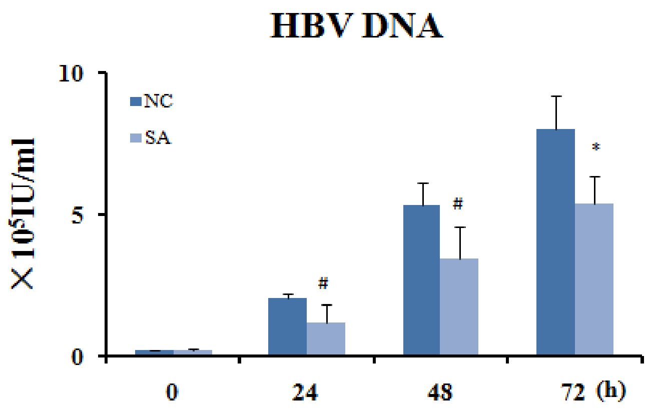

Supplement figure 7 Stearic acid inhibited HBV DNA secretion from 24 to 72 hours in HepG2.2.15 cells. HBV DNA in supernatants was determined by real-time polymerase chain reaction assay. NC, normal control. # P < 0.05 versus NC.
